# Efficacy of Mycotoxin Detoxifiers on Health and Growth of Newly-Weaned Pigs under Chronic Dietary Challenge of Deoxynivalenol

**DOI:** 10.3390/toxins12050311

**Published:** 2020-05-09

**Authors:** Debora Muratori Holanda, Sung Woo Kim

**Affiliations:** Department of Animal Science, North Carolina State University, Raleigh, NC 27695, USA; dmurato@ncsu.edu

**Keywords:** deoxynivalenol, health, yeast

## Abstract

The efficacy of yeast-based mycotoxin detoxifiers on health and growth performance of newly-weaned pigs (27-d-old) fed diets naturally contaminated with deoxynivalenol was investigated. Sixty pigs were individually assigned to five treatments for 34 d: NC (negative control, 1.2 mg/kg of deoxynivalenol); PC (positive control, 3.2 mg/kg of deoxynivalenol); CYC (PC + clay/yeast culture-based product, 0.2%); CYE (PC + clay/yeast cell wall/plant extracts/antioxidants-based product, 0.2%); and CYB (PC + clay/inactivated yeast/botanicals/antioxidants-based product, 0.2%). Blood and jejunal mucosa were sampled, and data were analyzed using Proc Mixed of SAS with pre-planned contrasts. Deoxynivalenol reduced the average daily gain (ADG) in phase 3. Pigs fed CYC had greater overall ADG, average daily feed intake during phase 3, and gain to feed ratio during phase 2 than PC. At d 14, deoxynivalenol reduced blood urea nitrogen/creatinine and tended to reduce blood urea nitrogen. Pigs fed CYB tended to have greater aspartate aminotransferase than PC. At d 34, pigs fed CYC and CYB tended to have lower serum creatine phosphokinase than PC. Pigs fed CYE had lower blood urea nitrogen/creatinine than PC. In jejunal mucosa, deoxynivalenol tended to increase malondialdehydes and decrease glutathione. Pigs fed CYE and CYB had lower malondialdehydes, pigs fed CYB had greater glutathione and tended to have lower immunoglobulin A than PC. Pigs fed CYC and CYE tended to have lower interleukin 8 than PC. In summary, deoxynivalenol challenge (1.2 vs. 3.2 mg/kg) mildly compromised growth performance and increased the oxidative stress of pigs. Mycotoxin detoxifiers could partially overcome deoxynivalenol toxicity enhancing liver health, whereas CYE and CYB reduced oxidative stress, and CYC and CYB reduced immune activation. In conclusion, yeast-based detoxifiers with functional components as clay/inactivated yeast/botanicals/antioxidants had increased detoxifying properties in newly-weaned pigs challenged with deoxynivalenol, potentially by enhancing adsorbability, immune function, gut health, and reducing oxidative stress.

## 1. Introduction

Mycotoxins are fungal metabolites that may have deleterious effects in animals [1]. Deoxynivalenol is a type B trichothecene produced by *Fusarium* species that may contaminate cereal grains used to formulate diets for livestock animals. Mycotoxins are detected in cereal grains worldwide, with a prevalence of 88% on feed and raw feedstuffs [2]. *Fusarium* toxins are the most prevalent globally, as well as in the United States, showing a higher frequency in corn and wheat and also occurring in byproducts of the food chain as bread/cookie meal, dried distillers grains with solubles (DDGS), and brewery wastes [2,3,4,5]. Corn is one of the main feedstuffs used in pig diets, where type B trichothecenes had on average 78% of occurrence and 1.235 mg/kg in samples from the United States in three years (2016–2018) [3]. Pigs are the most susceptible species to deoxynivalenol among domestic animals, in which deoxynivalenol can reduce feed intake, impair animal growth, trigger pro-inflammatory, and immunological responses, and even cause vomiting when in high concentrations in diets [6,7,8,9,10]. At the cellular level, deoxynivalenol induces a ribotoxic stress response (p38 mitogen-activated protein kinase, MAPK, activation by phosphorylation), regulating gene expression, by inhibiting translation, and triggering apoptosis, by the inhibition of protein elongation and the activation of apoptosis factors (nuclear factor-κB) [11,12]. Deoxynivalenol can also naturally occur as deoxynivalenol-3-glucoside, 3-acetyl-deoxynivalenol, 15-acetyl-deoxynivalenol, deoxynivalenol-3-sulfate, and deoxynivalenol-15-sulfate [13,14]. As extensively reviewed, although they are less toxic than deoxynivalenol, the deoxynivalenol modified forms can be converted to deoxynivalenol and then absorbed in the gastrointestinal tract of the pig, except for deoxynivalenol sulfates [12,13,15,16]. Thus, the modified forms may potentiate deoxynivalenol toxicity when present in animal feed. The co-contamination with other mycotoxins may influence deoxynivalenol kinetics and toxicity. For instance, another *Fusarium* toxin named culmorin can the hinder deoxynivalenol detoxification step of glucuronidation [17]. Therefore, the Food and Drug Administration stipulated advisory levels for not exceeding 1 mg/kg in diets for growing pigs [18]. Similarly, the deoxynivalenol limit in Europe for pigs is 0.9 mg/kg of feed [19]. Collectively, the high prevalence of deoxynivalenol, its toxicity for pigs, and the existing advisory levels make investigations for an effective mycotoxin deactivator valuable.

The use of mycotoxin detoxifiers as feed additives aims to reduce mycotoxin toxicity in contaminated feed ingredients, enabling their use for animal feed formulation [20]. There is a wide array of mycotoxin detoxifiers, with an equivalent myriad of claims. Mycotoxin detoxifiers may directly interact with deoxynivalenol molecules by the chemical transformation of deoxynivalenol to non-toxic compounds, or by the adsorbent properties of inorganic (clays) or organic substances (algal extracts, yeast, and yeast byproducts). However, it has been a great challenge to find interacting substances that can effectively counteract deoxynivalenol toxicity, because of the small chemical structure and low polarity of deoxynivalenol [10,21,22].

Mycotoxin detoxifiers that target deoxynivalenol often include components to promote gut health, stimulate the immune system, or provide sources of functional and conditionally essential nutrients (dietary fiber, plant derivatives, and nucleotides), combining different properties and improving detoxification ability [23]. Therefore, three yeast-based mycotoxin detoxifiers were chosen for evaluations under deoxynivalenol challenge in nursery pigs. First, CYC product (clay and yeast culture) is composed of bentonite clay, yeast culture (*Saccharomyces cerevisiae*, minimum 2.7 × 10^10^ CFU/kg), diatomaceous earth, and dehydrated kelp meal. Second, CYE product (clay, yeast, and plant extract) is composed of organo-aluminosilicate clays, a blend of hydrated sodium-calcium aluminosilicate clays, yeast cell walls, plant extracts, and antioxidants. The third, CYB product (clay, yeast, and botanicals) is composed of high adsorbent modified clay minerals, inactivated yeast, and fermentation extracts (*S. cerevisiae*), antioxidant and preservative mixture (calcium propionate), and botanicals (milk thistle, rosemary, licorice, and boldo).

Deoxynivalenol contamination in feedstuffs may range from 0 to 50 mg/kg, but it is most commonly lower than 5 mg/kg [24]. Specifically for naturally contaminated diets, the same study highlights that reductions in pig feed intake and growth are observed with more than 1 to 2 mg/kg, where an additional 1 mg/kg of deoxynivalenol results in an 8% decrease in pig growth [24]. Based on such outcomes, average levels in similar studies [6,8,10,25,26], and the advisory levels in the United States, the current study used diets naturally contaminated with an additional 2 mg/kg of deoxynivalenol (1.2 vs. 3.2 mg/kg). The first objective was to evaluate deoxynivalenol effects on health and growth performance in nursery pigs, due to the species susceptibility to deoxynivalenol, particularly at an early age. Second, to test the effects and efficacy of three yeast-based mycotoxin detoxifiers on reducing deoxynivalenol toxicity in pigs.

## 2. Results

**Figure 1:**
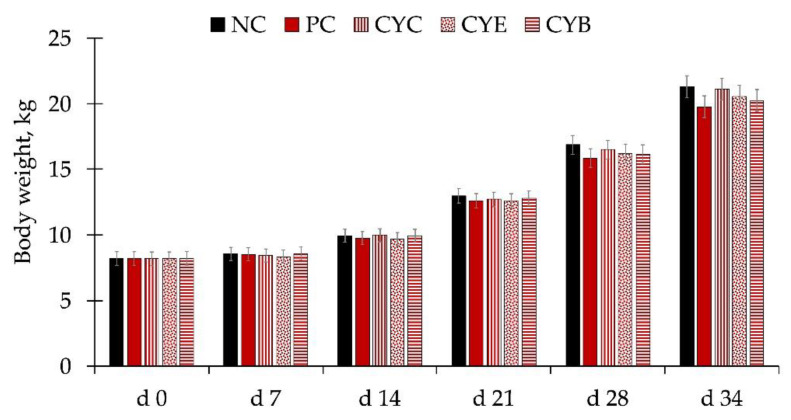
Body weight in newly-weaned pigs consuming diets with (PC, positive control diet with 3.2 mg/kg of deoxynivalenol) or without deoxynivalenol (NC, negative control diet with 1.2 mg/kg of deoxynivalenol) and diets with deoxynivalenol and mycotoxin detoxifiers at 0.2%: CYC (PC + clay/yeast culture-based product); CYE (PC + clay/yeast cell wall/plant extracts/antioxidants-based product); and CYB (PC + clay/inactivated yeast/botanicals/antioxidants-based product).

**Figure 2:**
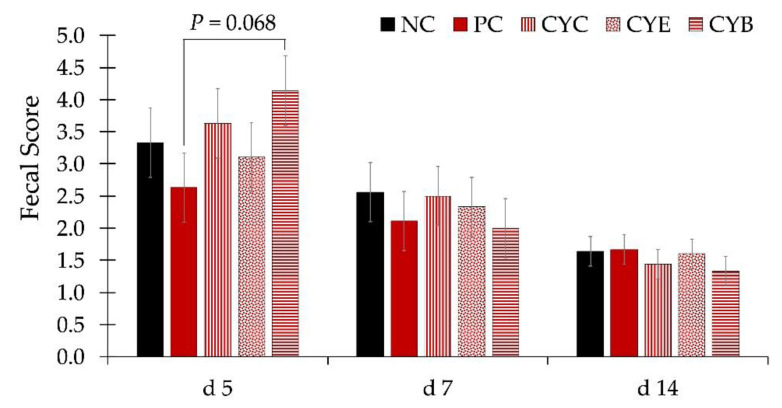
Fecal score in newly-weaned pigs consuming diets with (PC, positive control diet with 3.2 mg/kg of deoxynivalenol) or without deoxynivalenol (NC, negative control diet with 1.2 mg/kg of deoxynivalenol) and diets with deoxynivalenol and mycotoxin detoxifiers at 0.2%: CYC (PC + clay/yeast culture-based product); CYE (PC + clay/yeast cell wall/plant extracts/antioxidants-based product); and CYB (PC + clay/inactivated yeast/botanicals/antioxidants-based product). The fecal score was subjectively measured by a single evaluator based in a 1 to 5 scale, as described by Hu et al. [27].

The average high and low temperatures were 30.4 and 27.5 °C, with 74.6% specific humidity over the course of the whole experiment. There were no differences in the body weight of pigs among treatments during the whole experiment (Figure 1). Pigs fed PC (positive control, 3.2 mg/kg of deoxynivalenol) presented a tendency for lower average daily gain (ADG) from d 21 to 28 (*p* = 0.058) and d 28 to 34 (*p* = 0.087), and significantly lower (*p* < 0.05) during the whole phase 3 (d 21 to 34), in comparison to pigs fed NC (negative control, 1.2 mg/kg of deoxynivalenol; Table 1). Pigs fed PC presented a tendency (*p* = 0.099) for lower average daily feed intake (ADFI) from d 21 to 28, in comparison to pigs fed NC. Pigs fed PC showed a tendency for lower gain to feed ratio (G:F) during phase 2 (d 7 to 21; *p* = 0.066) and from d 14 to 21 (*p* = 0.087) in comparison to pigs fed NC. Pigs fed CYC (PC + CYC product at 0.2%) had greater (*p* < 0.05) ADG during phase 3 (d 21 to 34) and from d 28 to 34, than pigs fed PC. Pigs fed CYE (PC + CYE product at 0.2%) showed a higher fecal score than pigs fed PC on d 5 (Figure 2). There were no differences in the fecal score among pigs from experimental treatments on d 7 or 14.

Regarding the assessment of proteins, metabolites, and electrolytes in blood serum on d 14, pigs fed PC showed a tendency to lower (*p* = 0.078) blood urea nitrogen levels and lower (*p* < 0.05) blood urea nitrogen to creatinine ratio than pigs fed NC (Table 2). Pigs fed CYC showed a tendency (*p* = 0.056) for lower total protein and lower (*p* < 0.05) albumin levels than pigs fed PC. Pigs fed CYE showed a tendency for lower albumin (*p* = 0.086) and albumin to globulin ratio (*p* = 0.063) than pigs fed PC. Pigs fed CYB (PC + CYB product at 0.2%) had lower (*p* < 0.05) albumin, higher (*p* < 0.05) globulin, and lower (*p* < 0.05) albumin to globulin ratio than pigs fed PC. Pigs fed CYB showed a tendency (*p* = 0.086) for higher aspartate aminotransferase than pigs fed PC. Pigs fed CYB had a lower (*p* < 0.05) calcium concentration than pigs fed PC.

On d 34, a blood serum assessment of proteins, metabolites, and electrolytes showed that pigs fed CYC had higher glucose and tended to show lower creatine phosphokinase than pigs fed PC (Table 3). Pigs fed CYE had higher (*p* < 0.05) blood urea nitrogen to creatinine ratio than pigs fed PC. Pigs fed CYB showed a tendency for lower creatine phosphokinase (*p* = 0.071) and calcium (*p* = 0.051) than pigs fed PC.

Pigs fed PC tended to have a higher concentration of malondialdehydes (*p* = 0.069) and to have lower total glutathione (*p* = 0.067) in mid-jejunal mucosa, in comparison to pigs fed NC (Table 4). Pigs fed CYC tended to present lower (*p* = 0.079) interleukin 8 than pigs fed PC. Pigs fed CYE had lower (*p* < 0.05) malondialdehydes than pigs fed PC. Pigs fed CYB had lower (*p* < 0.05) malondialdehydes and higher (*p* < 0.05) glutathione than pigs fed PC. Pigs fed CYB tended to have lower (*p* = 0.081) immunoglobulin A than pigs fed PC. There were no differences in protein carbonyls, tumor necrosis factor-alpha, or immunoglobulin G in the mid-jejunal mucosa of pigs among experimental treatments.

**Figure 3:**
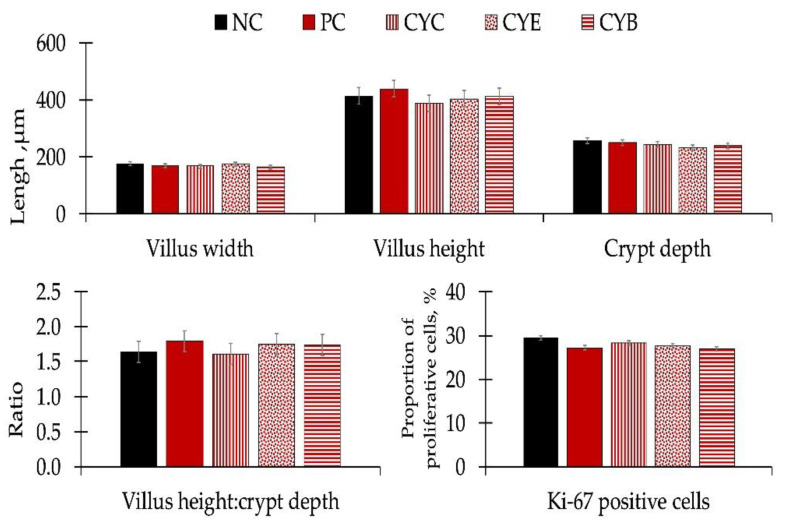
Intestinal morphology (top and bottom left graphs) and proportion of proliferative cells (bottom right) in the mid-jejunum sections of newly-weaned pigs consuming diets with (PC, positive control diet with 3.2 mg/kg of deoxynivalenol) or without deoxynivalenol (NC, negative control diet with 1.2 mg/kg of deoxynivalenol) and diets with deoxynivalenol and mycotoxin detoxifiers at 0.2%: CYC (PC + clay/yeast culture-based product); CYE (PC + clay/yeast cell wall/plant extracts/antioxidants-based product); and CYB (PC + clay/inactivated yeast/botanicals/antioxidants-based product) on d 34.

There were no differences in apparent ileal digestibility for dry matter, gross energy, nitrogen, and ether extract (Table 5), or for morphology or estimated proliferative rate of enterocytes in the crypt after Ki-67 staining performed in histology sections from the mid-jejunum (Figure 3) of pigs among experimental treatments. 

## 3. Discussion

The use of mycotoxin detoxifiers with multiple compositions has shown promising effects over mycotoxin detoxifiers with single components, notably when animals are challenged with deoxynivalenol [23]. Therefore, we tested three mycotoxin detoxifiers (CYC, CYE, and CYB) with multiple components in the current study. The chosen mycotoxin detoxifiers have in common variable sources of yeast. The CYC supplement is composed of bentonite, yeast culture (*S. cerevisiae*, minimum 2.7 × 10^10^ CFU/kg), diatomaceous earth, and dehydrated kelp meal. Bentonites have a weak binding ability in vitro for deoxynivalenol (3.24%), when compared to other mycotoxins, such as aflatoxins (92.5%) [28]. The lower adsorbability for deoxynivalenol in comparison to aflatoxins is probably due to the latter’s higher polarity [22,29]. However, in vivo, bentonites have shown the ability to reduce deoxynivalenol’s detrimental effect on bone mineralization in minks fed naturally contaminated diets with the mycotoxin [30]. The inclusion of yeast culture could potentiate bentonites effects by fermentation of sugars in the gastrointestinal tract yielding metabolites, with beneficial effects on the nutrition and health of pigs, like peptides and organic acids [31]. Indeed, yeast culture supplementation has shown improved total tract apparent digestibility, reduced *Escherichia coli* count in feces, and enhanced animal health, improving animal performance [32]. Diatomaceous earth has an intermediary binding ability to mycotoxins according to the review prepared by Huwig et al. [29], but its adsorbability is relatively high among mineral sources and it is among the ones with the highest adsorbability considering *Fusarium* toxins. The last component of CYC, kelp meal, may present antioxidant properties, being able to improve growth performance in broilers challenged with mycotoxins [33], as well as in pigs challenged with *E. coli* [34].

The CYE supplement is composed of organo-aluminosilicate clays and a blend of hydrated sodium-calcium aluminosilicate clays (sepiolite), yeast cell walls (from *S. cerevisiae*), plant extracts (a mixture of aromatic substances), triglycerides, calcium propionate, and antioxidants (citric acid, BHT, and ethoxyquin). The aluminosilicates have a relatively low adsorbing capacity to mycotoxins, but their adsorbability is greatly improved when in the hydrated sodium-calcium form, especially concerning aflatoxins [29], or when aluminosilicates are bound to organic components [35]. Yeast cell wall components, such as β-D-glucans and glucomannans, have *Fusarium* toxin adsorbing ability [36]. Indeed, processing yeast to yield yeast cell walls may enhance the binding ability to mycotoxins [29]. In porcine intestinal cells challenged with deoxynivalenol, the yeast cell wall could enhance enterocyte integrity and reduce inflammation [23]. In vivo studies with yeast cell wall as a unique additive to mitigate deoxynivalenol toxicity in pigs are scarce. The scarcity of publications is probably due to the limited effect of the yeast cell wall on improving animal health and growth in pigs challenged with deoxynivalenol [37,38], playing a minor role as a deoxynivalenol detoxifier. The plant extracts and antioxidants were included in this blend to promote gut health, by reducing the oxidative stress that may be provoked by mycotoxin challenge.

The CYB supplement is composed of high adsorbent modified clay minerals (sepiolite and bentonite), inactivated yeast and fermentation extracts (from *S. cerevisiae*), antioxidant (propyl gallate) and preservative mixture (calcium propionate extract), and botanicals (milk thistle seed, *Silybum marianum*; rosemary dried leaves, *Rosmarinus officinalis* L.; licorice root; and boldo dried leaves, *Peumus boldus*). These components have similar properties as the ones described for the first two mycotoxin detoxifiers. The yeast component in CYB can be roughly classified as an intermediary between CYC and CYE. The fermentation extracts may have similar beneficial effects of the yeast metabolites in CYC, but they will, rather, be present in the additive instead of being produced in the gut, because of using inactivated yeast. At the same time, the use of inactivated yeast may pose a role similar to CYE by enabling yeast cell wall interaction with enterocytes and with deoxynivalenol, enhancing gut health and acting as a mycotoxin detoxifier, respectively. The distinct component, in comparison to previous mycotoxin detoxifiers, is calcium propionate. Calcium propionate, as an organic acid, can lower the pH in the gastrointestinal tract, promoting gut health and nutrient digestibility [39]. Furthermore, calcium propionate is known to hinder fungal development in feed [40]. Regarding the mycotoxin challenge, calcium propionate could decrease mycotoxin accumulation in body tissues, improve liver health, and promote recovery in growth performance as shown in a study conducted with broilers [41]. In pigs, a mycotoxin detoxifier similar to CYB was tested against zearalenone, aflatoxin, and ochratoxin, showing promising detoxifying effects by improving gut health, nutrient digestibility and absorption, and animal growth [42].

In the current study, it was possible to notice the chronic toxicity of deoxynivalenol in pigs, after feeding deoxynivalenol contaminated diet for 21 d. When comparing pigs fed PC and NC, signals of deoxynivalenol impairment on growth performance started to be noticed after phase 2. For ADFI and ADG, deoxynivalenol toxicity was noticeable during phase 3, reducing both variables after d 21, when comparing pigs fed PC and NC. The higher fecal score observed for pigs fed CYE in comparison to pigs fed PC may suggest that pigs fed diets with CYE were more susceptible to deoxynivalenol toxicity in the gastrointestinal tract, resulting in a higher incidence of soft feces. The use of yeast cell walls, instead of the whole cell used in CYC and CYB, may have accounted for the increase in fecal score observed for CYE, but not to CYC and CYB. Ultimately, pigs fed CYC, showed greater G:F during phase 2 and ADG during phase 3 than pigs fed PC. This may suggest that the use of whole yeast cells or their fermentation products (CYC and CYB) may have induced gut health. Reinforcing such finding, pigs facing weaning stress supplemented with yeast culture showed increased IFN-γ production, suggesting cellular immunity recruitment in the gut, and eventual reduced lymphocyte T CD4+ activation [31,32]. Thus, the supposed increase in cellular immunity could have supported gut health post-weaning in CYC and CYB in the current study.

Feeding a deoxynivalenol contaminated diet with 3.2 mg/kg of feed did not show major impacts on growth performance in weaned pigs during 34 d. Deoxynivalenol is a mycotoxin known to depress animal feed intake [43] and may cause vomiting [7], resulting in impaired animal growth [44]. Indeed, in the review prepared by Pestka [11], reduced feed intake and growth are characteristics of deoxynivalenol chronic exposure, as observed in the current study, due to the upregulation of proinflammatory cytokines by p38 MAPK induced activation. Whereas vomiting is observed under acute exposure mediated by the increase in serotonin and peptide YY, where the latter also plays a role in appetite inhibition [7,43]. Considering that our research group has been performing related investigations, the number of replications and the mycotoxin concentration were expected to result in impaired animal growth [8,25,26,45,46]. Thus, it can be hypothesized that deoxynivalenol concentration in the NC diet (1 mg/kg of feed), could have impaired growth performance of pigs and was masking the outcomes observed in comparison to pigs fed PC. Supporting this hypothesis, it was observed in previous studies that deoxynivalenol concentrations of 0.6 mg/kg in naturally contaminated feed can impair the growth performance of pigs [8,24]. Thus, deoxynivalenol toxicity in PC treatment in comparison to NC was not able to elicit acute and marked differences in the growth performance of pigs. 

Unexpectedly, pigs fed PC had lower blood urea nitrogen and blood urea nitrogen to creatinine ratio than pigs fed NC on d 14. Nitrogen concentration in blood and its ratio to creatinine are related to liver and kidney health since both organs are the major sites of amino acid deamination, urea synthesis, and excretion. Pigs fed CYC presented lower total protein and albumin levels than pigs fed PC. Albumin is the major constituent of serum proteins; thus, its decrease may have led to the decrease observed in total protein. Similarly, pigs fed CYE and CYB presented lower albumin levels as well as for the ratio of albumin to globulin than pigs fed PC. The interaction of deoxynivalenol with the 60S ribosomal subunit hinders protein translation, also known as ribotoxic stress [12], which may have decreased serum protein concentration. In fact, albumin reduced synthesis was observed in pigs fed deoxynivalenol contaminated diet and such reductions were more pronounced after 28 days of deoxynivalenol challenge [47]. Likewise, the increase in aspartate aminotransferase observed in pigs fed CYB in comparison to PC is indicative of liver injury by leakage of the intracellular enzyme, since aspartate aminotransferase is the most sensible enzyme for liver injury in swine [48]. Deoxynivalenol also has a toxic effect on the kidney. The mycotoxin can impair kidney function by increasing serum creatinine and blood urea nitrogen, augment oxidative stress by increasing malondialdehydes and reducing superoxide dismutase activity, and eventually triggering renal cell apoptosis in mice [49]. There was a lower calcium concentration in serum of pigs fed CYB than pigs fed PC, suggesting that deoxynivalenol may have caused the hypocalcemia observed, due to its effects on the kidney. In addition, vitamin D deficiency related to liver malfunctioning induced by deoxynivalenol was related to lower calcium absorption leading to hypocalcemia [50]. Indeed, deoxynivalenol toxicity on kidney and liver health seems to have lasted until the end of the study on d 34 in pigs fed CYB, as seen for the lower calcium levels in comparison to pigs fed PC. On d 34, pigs fed CYC seemed to have improved liver and gut health, as seen by the lower creatine phosphokinase and higher glucose than pigs fed PC. As discussed previously, yeast culture metabolites provided by CYC may have improved gut integrity [31], then improving nutrient digestibility [32]. The improvement in liver health could be the eventual improvement by reduced mycotoxin absorption and its systemic toxicity observed at the liver. On the other hand, pigs fed CYE showed higher blood urea nitrogen to creatinine ratio than pigs fed PC, indicating impaired liver and kidney health. Such a result reinforces the increase observed in fecal score in pigs fed CYE in comparison to pigs fed PC, where there is an indication that yeast fermentation products may pose a bigger role in deoxynivalenol detoxification at the gastrointestinal level than the yeast cell wall alone.

The mid-jejunum (3.5 m distal from duodenum [51]) was targeted to evaluate the impacts of deoxynivalenol, because mid-jejunum is the major site for the absorption of deoxynivalenol [52] and the most important for nutrient absorption, and, thus, growth of pigs [53]. The tendency of an increase in malondialdehydes concentration, along with the decrease in total glutathione in pigs fed PC versus NC, is indicative of the fact that jejunal mucosa was under oxidative stress, due to a higher proportion of damaged cell components (malondialdehydes) and lower levels of antioxidant molecules (total glutathione). Similar results were found in another study, where deoxynivalenol intake culminated in increased malondialdehydes and decreased superoxide dismutase activity, another antioxidant enzyme [49]. Moreover, the glutathione-S-transferase plays a role in phase II detoxification of deoxynivalenol and, thus, depletion in total glutathione could be explained by its employment on for excretion of deoxynivalenol [12]. Nevertheless, such effects could not affect other oxidative stress markers, as protein carbonyls and immunological responses on tumor necrosis factor-alpha, interleukin 8, immunoglobulin A, and immunoglobulin G when comparing pigs fed NC and PC. Possibly, the chronic exposure to deoxynivalenol allowed the animals to overcome the mycotoxin toxicity and only mild effects could be observed after 34 days of exposure. Pigs fed the mycotoxin detoxifiers CYC and CYB presented lower interleukin 8 and lower immunoglobulin A, respectively, in comparison to pigs fed PC. In accordance with what was previously hypothesized, supplementation with *S. cerevisiae* either as yeast culture (CYC) or as inactivated yeast and fermentation extract (CYB) may have had an improved immune response. The yeast cell wall (CYE) did not show any immune-modulatory outcome in comparison to PC, suggesting that the whole cell was critical to trigger immunological effects in newly-weaned pigs challenged with deoxynivalenol in the current study. One yeast-derived component that may be absent in CYE that has previously shown immune-modulatory activity and improving liver health and gut function is nucleotides. Yeast-derived nucleotides have a role in gut development in pigs, increasing enzyme activity in the stomach (pepsin) and proximal small intestine (alkaline phosphatase) [54]. In the same study, nucleotide supplementation increased plasmatic immunoglobulin A and influenced lymphocyte proliferation in lymphoid tissue. Besides, nucleotide beneficial effects can be observed during rapid growth and, especially, in the case of liver injury [55], and may enhance hepatic glucuronidation [56] for further deoxynivalenol detoxification. Thus, such results indicate that yeast fermentation products may pose a bigger role as deoxynivalenol detoxifiers, by reducing immune response in pigs challenged with the mycotoxin. Additionally, pigs fed CYB showed lower malondialdehydes and higher total glutathione, explaining the reduced cell damage by increasing antioxidant molecules that can repair deoxynivalenol injuries at the cellular level or be used for deoxynivalenol clearance from the organism. The gut may host bacteria in charge of deoxynivalenol detoxification by de-epoxidation [57]. However, pig microbiome has a low ability to detoxify deoxynivalenol in comparison to other species [58] and most of it is restrained to the lower digestive tract, where the majority of deoxynivalenol intake was already absorbed [52,59]. The complete pathway of deoxynivalenol detoxification in the pig also involves hepatic metabolism [15]. The cytochrome P-450 takes the first detoxification phase in the liver for further processing by glutathione-S-transferase, glutathione peroxidase, catalase, or superoxide dismutase [60,61]. During phase II, the liver plays a controversial role in deoxynivalenol detoxification after oral intake. The major excretion route of deoxynivalenol is through urine (about 70%) in its intact form (95%) [62], with more significant detoxification occurring in the intestine [59,63], whereas other studies support hepatic glucuronidation, but with elevated individual variation [64,65]. Therefore, our results show that the maintenance of gut integrity is critical in pigs challenged with deoxynivalenol, rather than the additive’s binding ability. The presence of plant extracts and antioxidants in CYE and CYB may have accounted for the reduction in oxidative stress in the gut. Plant compositions (milk thistle, rosemary, licorice, and boldo) in CYB may reduce inflammation by decreasing tumor necrosis factor-alpha and, thus, increasing cell viability in vitro [66]. Particularly, rosemary or its extract have the proven ability to reduce oxidative stress, and inflammatory and immune activation, besides improving liver function under mycotoxin challenge [67,68]. Additionally, α- and β-D-glucans, as components of the yeast cell wall, may modulate immune function and selectively interact with the gut microbiome [69]. Such yeast cell wall effects may be potentiated by their properties in reducing oxidative stress [70,71], improving overall pig health.

The mild differences observed among pigs fed NC and pigs fed PC regarding oxidative stress markers and immunological responses are consistent with the results observed in the histomorphology of mid-jejunal sections and nutrient digestibility. Deoxynivalenol did not influence morphology or Ki-67 measurements performed in histology sections from the mid-jejunum of pigs among experimental treatments. Eventually, the maintenance in intestinal structure could conserve the apparent ileal digestibility for dry matter, gross energy, nitrogen, or ether extract in pigs among experimental treatments. 

In summary, feeding deoxynivalenol at 3.2 mg/kg of feed compromised ADG (reduced 14%), ADFI (tended to reduce by 12.3%), and G:F (tended to reduce by 10.7%) of newly-weaned pigs. Besides, pigs fed deoxynivalenol diets showed increased oxidative stress in mid-jejunum by increased malondialdehydes and reduced total glutathione. Yeast-based mycotoxin detoxifiers maintained growth performance and liver health, and improved intestinal health by reducing malondialdehydes (for clay/yeast/antioxidants), enhancing total glutathione (for clay/yeast/antioxidant/botanicals), and reducing immune activation (for clay/yeast/antioxidant/botanicals) in the mid-jejunum of pigs fed diets with deoxynivalenol at 3.2 mg/kg of feed.

## 4. Conclusions

In conclusion, feeding naturally contaminated diets with deoxynivalenol (1.2 vs. 3.2 mg/kg) reduced growth performance and increased gut oxidative stress in nursery pigs. Yeast-based mycotoxin detoxifiers improved gut health, but could not fully overcome toxic effects of deoxynivalenol diets regarding growth and liver health in nursery pigs, where the yeast-based mycotoxin detoxifier with functional components as mineral and organic adsorbents, antioxidants, immune modulators, and health and digestibility promoters, showed improvements on immune activation, and reduction in oxidative stress.

## 5. Materials and Methods

A protocol of this experiment was reviewed and approved by the Institutional Animal Care and Use Committee at North Carolina State University (Raleigh, NC, USA). The experiment was conducted at the North Carolina State University Metabolism Educational Unit (Raleigh, NC, USA).

On day zero, 30 barrows and 30 gilts weaned at 27 days of age (8.20 ± 0.10 kg) were allotted to 5 dietary treatments (*n* = 12), based on a completely randomized block design according to sex and body weight (heavy, medium, and light). Pigs were assigned to individual pens with metal screen floors, equipped with a nipple drinker and a feeder. Each pig received one of the following 5 diets for 34 days: (1) a diet with corn DDGS containing 1.2 mg/kg of deoxynivalenol (NC), (2) a diet with corn DDGS contaminated with deoxynivalenol to supplement 2 mg/kg deoxynivalenol (PC), (3) PC + clay/yeast culture based product, at 0.2% (CYC); (4) PC + clay/yeast cell wall/plant extracts/antioxidants based product, at 0.2% (CYE); and (5) PC + clay/inactivated yeast/botanicals/antioxidants based product, at 0.2% (CYB). Experimental diets were formulated to meet or exceed the nutrient requirements suggested by NRC (2012), following a 3-phase feeding program (Table 6). All experimental diets were sampled (from 9 different locations, totaling 2 kg per diet) and 200 g of each were sent to North Carolina Department of Agriculture (Raleigh, NC, USA) for proximate analysis, and another 200 g of each was sent to North Dakota State University Veterinary Diagnostic Laboratory (Fargo, ND, USA) for determination on mycotoxin concentration (Table 7. Feed ingredients, calculated, and analyzed composition of experimental diets with (PC) or without mycotoxins (NC), in a three-phase feeding program fed to newly-weaned pigs for 34 d, based on a 3-phase feeding program.). Mycotoxins were extracted from feed samples by acetonitrile:water (84:16, *v*/*v*), filtered, and then screened by liquid chromatography-mass spectrometry/mass spectrometry (LC-MS/MS) for mycotoxin detection. The NC diet had deoxynivalenol because corn DDGS used in the NC diet also included deoxynivalenol. However, the concentration of deoxynivalenol in PC still had 2 mg/kg more deoxynivalenol than NC, as planned.

The body weights of pigs and feed disappearances by pigs were recorded weekly to calculate average daily gain (ADG), average daily feed intake (ADFI), and gain to feed ratio (G:F). The pig fecal score was recorded on days 5, 7, and 14 [72]. On days 14 and 34, blood samples of were collected using 0.8 × 32 mm needles (Eclipse, Becton Dickinson Vacutainer Systems, Franklin Lakes, NJ, USA) from the jugular vein, in 10 mL collection tubes for blood serum (Becton Dickinson Vacutainer Systems, Franklin Lakes, NJ, USA). After clot development, collection tubes were centrifuged at 1509× *g* at 4 °C for 15 min (5811F, Eppendorf, Hamburg, HH, Germany). The supernatant, serum, was transferred to 1.5 mL tubes (Fisherbrand, Fisher Scientific, Hampton, NH, USA) and samples were stored at −80 °C in a freezer (812660-760, Thermo Fisher Scientific, Waltham, MA, USA). Serum samples were submitted to assess proteins, metabolites, and electrolytes at a commercial laboratory (Antech Diagnostic Laboratory, Cary, NC, USA).

At the end of the study, d 34, pigs were desensitized by penetrating captive bolt and euthanized by exsanguination of the vena cava. Intestinal samples were collected 3.5 m after the duodenum, delimited by the end of the anatomical association between duodenum and pancreas, and considered as mid-jejunum [51]. Samples of gut mucosa were scrapped from 15 cm sections from mid-jejunum and stored at −80 °C until laboratory analysis. Antioxidant status, immune response, and intestinal barrier function were evaluated in gut mucosa by quantifying protein carbonyls (STA-310, Cell Biolabs, Inc., San Diego, CA, USA), malondialdehydes (STA-330, Cell Biolabs, Inc., San Diego, CA, USA), total glutathione (STA-312, Cell Biolabs, Inc., San Diego, CA, USA), tumor necrosis factor-alpha (PTA00, R&D Systems, Inc., Minneapolis, MN, USA), interleukin 8 (P8000, R&D Systems, Inc., Minneapolis, MN, USA), immunoglobulin G (E100-104, Bethyl Laboratories, Inc., Montgomery, TX, USA), and immunoglobulin A (E100-102, Bethyl Laboratories, Inc., Montgomery, TX, USA). The protocols provided by the kit manufactures were followed for determining the relative concentrations to protein content (PierceTM BCA Protein Assay Kit, Thermo Fisher Scientific, Waltham, MA, USA) determined from the same samples.

Ileal digesta collected on d 34 was stored at −80 °C until processing for lab analysis: freeze-drying and grinding. Dried and ground ileal digesta samples were analyzed for the apparent ileal digestibility of dry matter [73], gross energy (6200 Calorimeter, Parr Instrument Company, Moline, IL, USA), nitrogen (AOAC method 990.03, [74]), and ether extract (AOAC method 920.39, [74]).

**Figure 4:**
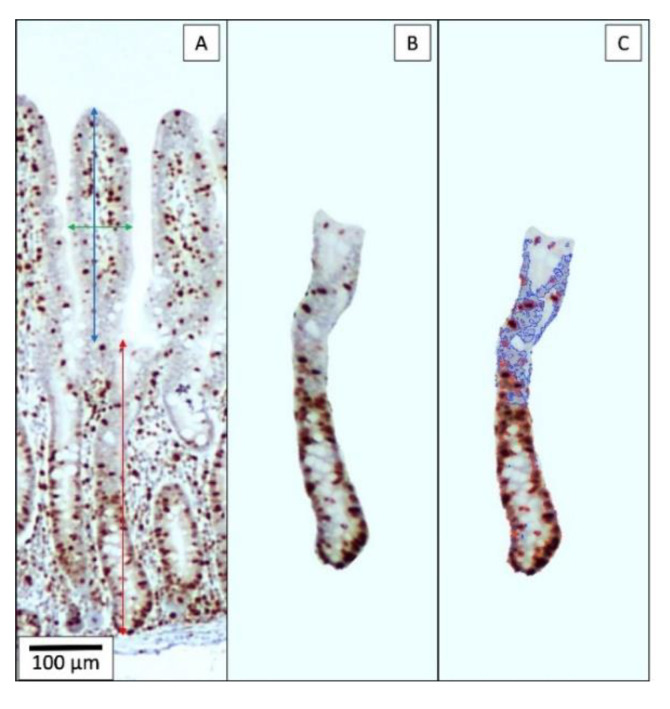
Ten microscopical images (40×) of a well-oriented villus and its associated crypt (**A**) were obtained for measuring villus height (double arrow line in blue), villus width (double arrow line in green), and crypt depth (double arrow line in red). This was followed by cropping the crypt (**B**) and assessing the nuclei of cells positive to Ki-67 staining (delimited in red, **C**) in proportion to total cell number (sum of nuclei delimited in blue and red). (**C**) was obtained using ImageJS tool [76].

A fragment of gut tissue (5 cm) from mid-jejunum was fixed in 10% buffered formalin for histological evaluation. Transversal sections from mid-jejunum were sent to the North Carolina State University Histopathology Laboratory (College of Veterinary Medicine, Raleigh, NC, USA) for inclusion in paraffin and immunohistochemistry staining for Ki-67 antigen [75]. The evaluation of mid-jejunal morphology in histological sections was assessed by one evaluator by measuring villus height and width, crypt depth, villus height:crypt depth ratio, and for estimating the percentage of proliferating cells in the crypt after Ki-67 staining [76] in ten pictures of each pig (Figure 4). The percentage of proliferating cells is calculated by dividing the nuclear area of cells positive to the Ki-67 antigen by the nuclear area of all cells in the crypt.

The statistical analysis was performed by using the MIXED procedure of SAS 9.3 software (Statistical Analysis System, Cary, NC, USA, 2011). The experimental unit was considered as a pen (one pig). Blocks (sex and initial body weight) were considered as random effects. The effect of sex was checked by assessing the interaction among treatments and sex using the MIXED procedure, but no effect was observed. Analyses of pre-planned contrasts between NC and PC as well as PC and mycotoxin detoxifiers (CYC, CYE, and CYB) were performed using the CONTRAST statement for comparisons with *F* test. The LSMEANS statement was used for the separation of means. Results were considered statistically different for *p* < 0.05 and 0.05 ≤ *p* < 0.10 were considered tendency. The design was based on the power test using previous studies conducted under similar measures and objectives (*n* = 12 was the minimum, considering a large individual variation).

## Figures and Tables

**Table 1:** Animal performance variables in newly-weaned pigs consuming diets with (PC ^1^) or without deoxynivalenol (NC) and diets with deoxynivalenol and mycotoxin detoxifiers ^2^.

Treatment	NC	PC	Additive			SEM	*p* Value			
			CYC	CYE	CYB		NC vs. PC	PC vs. CYC	PC vs. CYE	PC vs. CYB
ADG ^3^, g/d										
Phase 1 (d 0 to 7)	49	47	35	23	52	26	0.939	0.668	0.399	0.868
Phase 2 (d 7 to 21)	316	291	303	303	301	25	0.416	0.705	0.708	0.752
d 7 to 14	199	176	218	191	191	26	0.479	0.205	0.648	0.632
d 14 to 21	434	407	389	415	411	30	0.493	0.651	0.835	0.920
Phase 3 (d 21 to 34)	641	551	649	615	573	31	0.033	0.024	0.124	0.592
d 21 to 28	555	465	544	517	480	34	0.058	0.102	0.267	0.743
d 28 to 34	740	651	771	729	681	40	0.087	0.026	0.132	0.561
Overall (d 0 to 34)	385	340	380	365	354	22	0.106	0.162	0.377	0.627
ADFI ^4^, kg/d										
Phase 1 (d 0 to 7)	115	124	114	112	100	21	0.649	0.602	0.525	0.223
Phase 2 (d 7 to 21)	394	391	371	388	387	31	0.951	0.559	0.915	0.892
d 7 to 14	261	246	252	243	248	29	0.592	0.828	0.937	0.942
d 14 to 21	526	537	491	532	526	39	0.809	0.321	0.913	0.805
Phase 3 (d 21 to 34)	954	852	940	904	857	50	0.168	0.161	0.396	0.930
d 21 to 28	800	702	788	769	732	46	0.099	0.157	0.253	0.613
d 28 to 34	1,096	1,027	1,118	1,060	1,004	61	0.374	0.253	0.664	0.768
Overall (d 0 to 34)	544	512	536	528	508	34	0.393	0.531	0.667	0.897
G:F ^5^										
Phase 1 (d 0 to 7)	0.35	0.14	0.27	0.31	0.25	0.22	0.460	0.656	0.746	0.688
Phase 2 (d 7 to 21)	0.81	0.74	0.82	0.79	0.77	0.03	0.066	0.045	0.217	0.399
d 7 to 14	0.76	0.72	0.88	0.68	0.76	0.08	0.749	0.178	0.674	0.742
d 14 to 21	0.84	0.75	0.80	0.80	0.78	0.03	0.087	0.329	0.310	0.615
Phase 3 (d 21 to 34)	0.68	0.66	0.69	0.68	0.67	0.03	0.406	0.372	0.261	0.749
d 21 to 28	0.70	0.67	0.69	0.68	0.65	0.03	0.425	0.544	0.622	0.717
d 28 to 34	0.68	0.66	0.69	0.70	0.68	0.04	0.603	0.463	0.302	0.537
Overall (d 0 to 34)	0.71	0.67	0.71	0.70	0.69	0.02	0.112	0.145	0.236	0.330

^1^ PC diets have 2 mg/kg of deoxynivalenol supplemented from mycotoxin contaminated corn DDGS; ^2^ Mycotoxin detoxifiers were added to PC at 0.2% in all phases to create three other dietary treatments: CYC (PC + clay/yeast culture based product); CYE (PC + clay/yeast cell wall/plant extracts/antioxidants based product); and CYB (PC + clay/inactivated yeast/botanicals/antioxidants based product); ^3^ ADG, average daily gain; ^4^ ADFI, average daily feed intake; ^5^ G:F, gain to feed ratio.

**Table 2:** Serum variables for proteins, metabolites, and electrolytes in newly-weaned pigs consuming diets with (PC) or without deoxynivalenol (NC) and diets with deoxynivalenol and mycotoxin detoxifiers on d 14.

Treatment	NC	PC	Additive			SEM	*p* Value			
			CYC	CYE	CYB		NC vs. PC	PC vs. CYC	PC vs. CYE	PC vs. CYB
Total protein, g/dL	4.65	4.66	4.45	4.62	4.54	0.11	0.937	0.056	0.695	0.275
Albumin, g/dL	3.05	3.16	2.90	2.98	2.85	0.07	0.283	0.015	0.086	0.003
Globulin, g/dL	1.60	1.50	1.55	1.63	1.69	0.10	0.265	0.600	0.139	0.036
Albumin/globulin	1.98	2.15	1.91	1.87	1.74	0.13	0.268	0.115	0.063	0.008
AST ^1^, IU/L	47.08	36.50	46.00	43.00	50.25	5.18	0.147	0.202	0.370	0.062
ALT ^2^, IU/L	17.92	17.08	16.90	17.58	16.83	0.99	0.461	0.876	0.658	0.825
ALP ^3^, IU/L	222.6	205.7	231.0	216.3	205.7	17.8	0.416	0.235	0.607	1.000
BUN ^4^, mg/dL	14.25	12.25	11.69	10.50	12.17	0.97	0.078	0.621	0.121	0.941
Creatinine, mg/dL	0.93	0.94	0.89	0.86	0.88	0.07	0.761	0.389	0.132	0.289
BUN/creatinine	15.83	13.17	13.43	12.42	14.00	1.29	0.031	0.829	0.535	0.491
Glucose, mg/dL	79.83	79.58	82.08	75.33	78.83	6.65	0.968	0.697	0.498	0.905
Cholesterol, mg/dL	66.50	72.08	72.35	79.25	71.08	6.71	0.447	0.972	0.330	0.891
CPK ^5^, IU/L	2982	1598	2361	1590	1607	670	0.147	0.430	0.993	0.992
Phosphorus, mg/dL	9.26	9.32	9.69	9.53	9.73	0.44	0.870	0.311	0.543	0.254
Calcium, mg/dL	9.73	9.82	9.55	9.63	9.39	0.24	0.678	0.192	0.341	0.038
Sodium, mEq/L	143.9	143.7	144.5	143.7	144.0	1.2	0.835	0.511	1.000	0.782
Potassium, mEq/L	6.19	6.19	6.29	6.35	6.21	0.24	1.000	0.744	0.640	0.961
Sodium/potassium	23.75	23.58	23.27	22.92	23.67	0.86	0.891	0.803	0.584	0.945
Chloride, mEq/L	105.4	106.3	106.4	105.3	105.9	0.8	0.461	0.921	0.376	0.767

^1^ AST, aspartate amino transferase; ^2^ ALT, alanine amino transferase; ^3^ ALP, alkaline phosphatase; ^4^ BUN, blood urea nitrogen; ^5^ CPK, creatine phospho-kinase.

**Table 3:** Serum variables for proteins, metabolites, and electrolytes in newly-weaned pigs consuming diets with (PC) or without deoxynivalenol (NC) and diets with deoxynivalenol and mycotoxin detoxifiers on d 34.

Treatment	NC	PC	Additive			SEM	*p* Value			
			CYC	CYE	CYB		NC vs. PC	PC vs. CYC	PC vs. CYE	PC vs. CYB
Total protein, g/dL	4.88	4.78	4.58	4.77	4.66	0.10	0.462	0.109	0.892	0.312
Albumin, g/dL	3.11	3.06	2.91	3.03	2.86	0.09	0.674	0.188	0.821	0.075
Globulin, g/dL	1.77	1.73	1.67	1.73	1.80	0.07	0.604	0.571	0.929	0.424
Albumin/globulin	1.76	1.80	1.80	1.78	1.63	0.09	0.778	1.000	0.895	0.190
AST, IU/L	38.18	41.00	35.45	37.92	33.42	4.55	0.665	0.395	0.628	0.236
ALT, IU/L	20.82	22.83	21.09	21.92	20.58	1.57	0.364	0.435	0.673	0.303
ALP, IU/L	244.4	237.4	265.2	253.8	240.2	16.6	0.775	0.203	0.439	0.897
BUN, mg/dL	0.15	0.11	0.10	0.10	0.14	0.02	0.347	0.832	0.829	0.388
Creatinine, mg/dL	13.6	12.5	11.7	13.4	12.5	1.2	0.288	0.455	0.411	1.000
BUN/creatinine	0.77	0.74	0.71	0.67	0.71	0.04	0.548	0.505	0.138	0.506
Glucose, mg/dL	17.6	17.1	16.3	21.4	17.9	1.57	0.758	0.695	0.028	0.665
Cholesterol, mg/dL	102.6	93.3	107.6	100.2	99.7	4.2	0.124	0.020	0.246	0.282
CPK, IU/L	64.91	65.00	65.16	66.42	65.08	3.80	0.981	0.973	0.763	0.986
Phosphorus, mg/dL	11.10	10.58	10.58	10.96	10.82	0.37	0.182	0.986	0.299	0.517
Calcium, mg/dL	9.84	9.89	9.91	9.93	9.53	0.13	0.763	0.925	0.853	0.051
Sodium, mEq/L	139.4	137.8	138.5	139.1	138.0	0.8	0.139	0.495	0.213	0.867
Potassium, mEq/L	5.56	5.40	5.54	5.62	5.58	0.19	0.544	0.613	0.412	0.507
Sodium/potassium	25.64	25.75	25.18	24.92	25.00	0.79	0.920	0.614	0.450	0.497
Chloride, mEq/L	100.5	99.0	100.3	99.8	99.3	0.7	0.109	0.148	0.345	0.776

**Table 4:** Inflammatory response and oxidative stress markers quantification from mid-jejunal mucosa in newly-weaned pigs consuming diets with (PC) or without deoxynivalenol (NC) and diets with deoxynivalenol and mycotoxin detoxifiers on d 34.

Treatment	NC	PC	Additive			SEM	*p* Value			
			CYC	CYE	CYB		NC vs. PC	PC vs. CYC	PC vs. CYE	PC vs. CYB
Concentration/mg of protein										
Protein carbonyl, nmol	3.608	3.190	4.343	2.770	3.886	0.748	0.660	0.238	0.659	0.464
Malondialdehyde, μM	0.404	0.619	0.452	0.305	0.371	0.108	0.069	0.164	0.009	0.037
Total glutathione, μM	4.274	2.594	2.424	3.232	4.439	0.641	0.067	0.854	0.481	0.045
TNF-α ^1^, pg	1.091	1.196	0.859	1.031	1.190	0.243	0.759	0.356	0.630	0.984
IL-8 ^2^, ng	0.507	0.661	0.468	0.486	0.513	0.076	0.150	0.079	0.102	0.166
IgA ^3^, μg	2.610	4.032	3.528	2.752	2.284	0.734	0.154	0.618	0.198	0.081
IgG ^4^, μg	2.169	1.657	1.710	1.811	2.144	0.309	0.242	0.905	0.722	0.266

^1^ TNF-α, tumor necrosis factor alpha; ^2^ IL-8, interleukin 8; ^3^ IgA, immunoglobulin A; ^4^ IgG, immunoglobulin G.

**Table 5:** Apparent ileal digestibility in newly-weaned pigs consuming diets with (PC) or without deoxynivalenol (NC) and diets with deoxynivalenol and mycotoxin detoxifiers on d 34.

Treatment	NC	PC	Additive			SEM	*p* Value			
			CYC	CYE	CYB		NC vs. PC	PC vs. CYC	PC vs. CYE	PC vs. CYB
Dry matter, %	61.92	53.31	43.86	51.61	55.95	6.87	0.189	0.160	0.794	0.608
Gross energy, %	59.67	52.07	51.77	57.27	56.59	6.25	0.248	0.965	0.429	0.491
Nitrogen, %	70.80	64.76	67.51	69.62	67.34	5.42	0.203	0.569	0.305	0.584
Ether extract, %	96.64	95.79	95.74	96.50	96.21	0.76	0.372	0.961	0.456	0.635

**Table 6:** Concentrations of detectable mycotoxins in conventional dried distillers grains with solubles (DDGS) or high in deoxynivalenol (DON) contamination DDGS used for diet formulation to newly-weaned pigs for 34 d, based on a 3-phase feeding program.

Mycotoxin, μg/kg	Corn DDGS	
	Conventional	High in DON
Aflatoxin B1	5.5	7.4
Aflatoxin B2	ND	ND
Aflatoxin G1	0.1	0.1
Aflatoxin G2	4.5	3.3
Ochratoxin A	2.0	2.1
Ochratoxin B	ND	1.7
Citrinin	57.0	88.0
Deoxynivalenol	2064	5643
3-acetyl-deoxynivalenol	21.7	97.0
15-acetyl-deoxynivalenol	550	2,009
Deoxynivalenol-3-glucoside	38.7	61.5
Nivalenol	5540	ND
Fusarenon-X	158	142
Beauvericin	1.0	0.4
Moniliformin	92.1	59.6
Fusaric acid	431	456
T2 toxin	6.0	1.7
HT2 toxin	100	254
Diacetoxyscirpenol	4.1	6.9
Neosolaniol	3.2	1.5
Fumonisin B1	479	199
Fumonisin B2	27.3	26.9
Fumonisin B3	5.0	9.7
Zearalenone	213	2,417
Patulin	17.5	9.9
Alternariol	21.2	47.4
Citreoviridin	2.1	2.9
Cyclopiazonic acid	2.7	2.5
Ergocornin	2.5	1.9
Ergocristine	145	100
Ergocryptine	26.1	115
Ergometrine	0.0	0.0
Ergosine	38.5	70.2
Ergotamine	217	242
Gliotoxin	438	2468
Lysergol	0.1	ND
Methylergonovine	0.5	0.7
Mycophenolic acid	0.3	0.7
Penicillic acid	7.0	5.0
Roquefortine C	0.2	0.3
Sterigmatocystin	1.0	0.9
Verruculogen	11.3	53.7
Wortmannin	17.7	8.7

Mycotoxin concentrations were measured at 37+ Lab (Alltech Inc., Nicholasville, KY, USA). ND, not detected.

**Table 7:** Feed ingredients, calculated, and analyzed composition of experimental diets with (PC) or without mycotoxins (NC), in a three-phase feeding program fed to newly-weaned pigs for 34 d, based on a 3-phase feeding program.

Item	Phase 1 (d 0 to 7)		Phase 2 (d 7 to 21)		Phase 3 (d 21 to 34)	
	NC	PC	NC	PC	NC	PC
Ingredient, %						
Ground corn	14.67	14.67	31.07	31.07	43.42	43.42
Corn DDGS	22.00	-	22.00	-	22.00	-
Corn DDGS with DON ^1^	-	22.00	-	22.00	-	22.00
Soybean meal	16.00	16.00	19.00	19.00	30.00	30.00
Whey permeate	20.00	20.00	10.00	10.00	-	-
Cookie meal	10.00	10.00	5.00	5.00	-	-
Poultry meal	6.00	6.00	4.00	4.00	-	-
Blood plasma	5.00	5.00	3.00	3.00	-	-
Fish meal	2.00	2.00	-	-	-	-
Poultry fat	2.00	2.00	3.00	3.00	2.00	2.00
Limestone	0.90	0.90	1.05	1.05	1.15	1.15
Dicalcium phosphate	-	-	0.50	0.50	0.70	0.70
Salt	0.22	0.22	0.22	0.22	0.22	0.22
L-lysine HCl	0.53	0.53	0.51	0.51	0.30	0.30
DL-methionine	0.15	0.15	0.12	0.12	0.02	0.02
L-threonine	0.10	0.10	0.10	0.10	0.01	0.01
Mineral mix	0.15	0.15	0.15	0.15	0.15	0.15
Vitamin mix	0.03	0.03	0.03	0.03	0.03	0.03
Titanium dioxide	-	-	-	-	0.05	0.05
Calculated composition						
DM, %	91.10	91.10	90.48	90.48	89.57	89.57
ME, kcal/kg	3471	3471	3480	3480	3391	3391
SID Lys, %	1.504	1.504	1.349	1.349	1.228	1.228
SID Thr, %	0.876	0.876	0.800	0.800	0.732	0.732
SID Trp, %	0.246	0.246	0.221	0.221	0.232	0.232
SID Met+Cys, %	0.817	0.817	0.744	0.744	0.680	0.680
Ca, %	0.849	0.849	0.799	0.799	0.711	0.711
STTD P, %	0.469	0.469	0.407	0.407	0.334	0.334
Analyzed mycotoxin ^2^, mg/kg						
Zearalenone	0.179	0.355	0.156	0.244	ND	0.266
Deoxynivalenol	1.262	3.015	1.265	3.027	1.131	3.561
Fumonisin B1	ND	ND	ND	ND	0.238	0.203

NC, negative control diet formulated with DDGS contaminated with 2.6 mg/kg of deoxynivalenol; PC, positive control diet formulated with DDGS contaminated with 7.6 mg/kg of deoxynivalenol; ND, not detected. ^1^ DON corn DDGS, deoxynivalenol contaminated corn DDGS (mycotoxin concentration: 7.6 mg/kg of feed of deoxynivalenol, 0.2 mg/kg of feed of fumonisin B1, and 0.5 mg/kg of feed of fusaric acid). ^2^ Mycotoxin concentrations were measured by LC-MS/MS Screen at the North Dakota State University Veterinary Diagnostic Laboratory (Fargo, ND, USA). Ingredients that were not included in a given formula have a dash (-) on its respective column.
